# *Staphylococcus warneri* dampens SUMOylation and promotes intestinal inflammation

**DOI:** 10.1080/19490976.2024.2446392

**Published:** 2025-01-16

**Authors:** Léa Loison, Marion Huré, Benjamin Lefranc, Jérôme Leprince, Christine Bôle-Feysot, Moïse Coëffier, David Ribet

**Affiliations:** a Univ Rouen Normandie, INSERM, Normandie Univ, ADEN, UMR 1073 Nutrition, Inflammation and Microbiota-Gut-Brain axis, Rouen, France; b Univ Rouen Normandie, INSERM, Normandie Univ, NorDiC, UMR 1239, PRIMACEN, Rouen, France; c Univ Rouen Normandie, INSERM, Normandie Univ, ADEN, UMR 1073 Nutrition, Inflammation and Microbiota-Gut-Brain axis, CHU Rouen, Department of Nutrition, CIC-CRB1404, Rouen, France

**Keywords:** Gut microbiota, *Staphylococcus warneri*, Warnericin RK, SUMOylation, host–bacteria interactions, inflammation, Ubiquitin-like modification

## Abstract

Gut bacteria play key roles in intestinal physiology, via the secretion of diversified bacterial effectors. Many of these effectors remodel the host proteome, either by altering transcription or by regulating protein post-translational modifications. SUMOylation, a ubiquitin-like post-translational modification playing key roles in intestinal physiology, is a target of gut bacteria. Mutualistic gut bacteria can promote SUMOylation, via the production of short- or branched-chain fatty acids (SCFA/BCFA). In contrast, several pathogenic bacteria were shown to dampen SUMOylation in order to promote infection. Here, we demonstrate that *Staphylococcus warneri*, a natural member of the human gut microbiota, decreases SUMOylation in intestinal cells. We identify that Warnericin RK, a hemolytic toxin secreted by *S. warneri*, targets key components of the host SUMOylation machinery, leading to the loss of SUMO-conjugated proteins. We further demonstrate that Warnericin RK promotes inflammation in intestinal and immune cells using both SUMO-dependent and SUMO-independent mechanisms. We finally show that Warnericin RK regulates the expression of genes involved in intestinal tight junctions. Together, these results highlight the diversity of mechanisms used by bacteria from the gut microbiota to manipulate host SUMOylation. They further highlight that changes in gut microbiota composition may impact intestinal inflammation, by altering the equilibrium between bacterial effectors promoting or dampening SUMOylation.

## Introduction

The gut microbiota plays essential roles in host physiology.^1^ Metabolites produced by intestinal microorganisms are essential mediators of host–microbiota interactions.^2^ They may regulate host cell activities by activating cell signaling pathways, by regulating cell transcription or by modulating host post-translational modifications.^3^ Short-chain fatty acids (SCFAs) and branched-chain fatty acids (BCFAs), for example, were shown to participate in the maintenance of intestinal epithelial integrity and to dampen inflammation by promoting intestinal cell SUMOylation.^4,5^

SUMOylation is a post-translational modification consisting in the covalent addition of a small polypeptide, named SUMO (for small ubiquitin-like modifier), to target proteins.^6,7^ The human genome codes for five SUMO paralogs that share 45–97% sequence homology. SUMO1, SUMO2 and SUMO3 display ubiquitous expression patterns and are the most widely studied paralogs (SUMO4 and SUMO5 expression being restricted to only a few, non-intestinal, tissues).^7^ SUMO2 and SUMO3 share 97% peptide sequence identity and are often referred to as SUMO2/3. SUMO1 and SUMO2/3 are not functionally redundant and are conjugated to distinct yet overlapping sets of proteins.^8^ Conjugation of SUMO to target proteins occurs on lysine side-chains and requires an enzymatic machinery composed of an E1 enzyme (formed by the SAE1/SAE2 heterodimer), an E2 enzyme (Ubc9) and E3 enzymes.^9^ SUMOylation is a reversible mechanism. Indeed, several proteases, called deSUMOylases, can cleave the covalent bond between SUMO peptides and their targets.^10^ Conjugation of SUMO may have multiple consequences, including changes in protein activity, stability, localization, or interactions with other cellular components.^6,7,11^ SUMOylation is more particularly involved in immunity and in intestinal homeostasis, as it limits detrimental inflammation and participates in tissue integrity maintenance.^4,12–15^

In contrast to mutualistic bacteria producing SCFAs and BCFAs, which increase intestinal SUMOylation, some pathogenic bacteria were shown to interfere with host SUMOylation in order to promote infection.^16–18^ The food-borne pathogen *Listeria monocytogenes*, for example, secretes a pore-forming toxin called Listeriolysin O (LLO), which triggers a rapid degradation of the SUMO E2 enzyme, thereby inhibiting *de novo* SUMO-conjugations in host cells.^19^ As deSUMOylases are still active in this case, this leads to a decrease in the level of SUMO-conjugated proteins in cells exposed to LLO, which has been correlated to an increase in bacterial infection.^19,20^ Other pathogens were shown to target the host SUMOylation machinery such as *Shigella flexneri*, which induces a calpain-dependent cleavage of the SUMO E1 enzyme SAE2, or *Salmonella enterica* serovar Typhimurium, which downregulates the level of SUMO E2 enzyme using miRNA-dependent mechanisms.^21,22^ *Staphylococcus aureus* was also shown to target the SUMO E2 enzyme in macrophages in order to promote intracellular survival.^23^

In contrast to pathogens, the impact of opportunistic pathogens on host SUMOylation is less documented. In this study, we focused on *Staphylococcus warneri*, a coagulase-negative staphylococci (CoNS) species naturally present in the human skin and gut microbiota.^24^ This bacterium has rarely been associated with infections and has a lower pathogenic potential than other CoNS such as *S. epidermidis*.^25^ Interestingly, *S. warneri* was recently shown to be able to invade intestinal cells.^24^ Here, we investigate whether *S. warneri* can interfere with host SUMOylation, similarly to *bona fide* intracellular pathogens such as *L. monocytogenes*, *S. flexneri* or *S*. Typhimurium.

## Material and methods

### Cell culture

Human Caco2 (American Type Culture Collection (ATCC) HTB-37) and HeLa (ATCC CCL-2) cells were cultivated at 37°C in a 5% CO_2_ atmosphere in Minimum Essential Medium (MEM) supplemented with 10% fetal bovine serum (FBS), 2 mM L-glutamine (Invitrogen), non-essential aminoacids (Sigma-Aldrich), 1 mM sodium pyruvate (Gibco) and a mixture of penicillin (10000 U/mL) and streptomycin (10 mg/mL). Murine RAW264.7 macrophages (ATCC TIB-71) were cultivated at 37°C in a 5% CO_2_ atmosphere in Dulbecco’s Modified Eagle’s Medium (DMEM) supplemented with 10% fetal bovine serum (FBS), 2 mM L-glutamine (Invitrogen) and a mixture of penicillin (10000 U/mL) and streptomycin (10 mg/mL). Human HIEC-6 (ATCC CRL-3266) were cultivated at 37°C in a 5% CO_2_ atmosphere in Opti-MEM^TM^ I Reduced Serum Medium (Gibco) supplemented with 4% FBS, 20 mM HEPES buffer (MP Biomedicals), 10 mM GlutaMAX^TM^ (Gibco) and 10 ng/mL Epidermal Growth Factor (EGF) recombinant human protein (Gibco). Cell viability was quantified using the CellTiter-Glo® Luminescent Cell Viability Assay (Promega).

### Bacterial strains

The bacterial strains used in this study were *Staphylococcus warneri* AW25 type strain (DSM 20316, DSMZ, Germany), *Staphylococcus epidermidis* ATCC 14990 type strain (DSM 20044, DSMZ, Germany) and *Escherichia coli* BL21(DE3)pLysS strain. Staphylococcal strains and *Escherichia coli* were grown at 37°C in, respectively, brain heart infusion (BHI) or LB broth or agar plates (BD Biosciences).

### Production of bacterial toxins

Warnericin RK (MQFITDLIKKAVDFFKGLFGNK) and delta-lysin II (MTADIISTIGDFVKWILDTVKKFTK) peptides, as well as their N-terminal formylated counterparts, were synthesized by Fmoc solid-phase methodology on a Liberty microwave assisted automated peptide synthesizer (CEM, Saclay, France), using the standard manufacturer’s procedures at 0.1 mmol scale as previously described.^26^ All Fmoc-amino acids (0.5 mmol, 5 eq.) were coupled on Fmoc-Lys(Boc)-HMP resin, by *in situ* activation with HBTU (0.5 mmol, 5 eq.) and DIEA (1 mmol, 10 eq.) before Fmoc removal with a 20% piperidine in DMF. After completion of the chain assembly, the formulation of the N-terminal extremity was carried out on solid support, as previously described.^27^ Briefly, the formylating agent was first prepared by mixing formic acid (25 mmol, 500 eq.) and diisopropylcarbodiimide (12.5 mmol, 250 eq.) in diethyl ether (14.5 mL) for 4 h at 0°C. Then, after filtration and concentration under vaccum, the obtained solution (2 mL) was added to the peptidyl-resin (0.05 mmol) in 1 mL of DMF with DIEA (0.12 mmol, 2,4 eq.) and kept at 4°C overnight, followed by DMF and DCM washes of the resin. The two peptides and their formylated counterparts were deprotected and cleaved from the resin by adding 10 mL of the mixture TFA/TIS/H2O (9.5:0.25:0.25) for 180 min at room temperature. After filtration, crude peptides were washed twice by precipitation in TBME followed by centrifugation (4500 rpm, 15 min). The synthetic peptides were purified by the reversed-phase HPLC (Gilson, Villiers le Bel, France) on a 21.2 × 250 mm Jupiter C18 (5 µm, 300 Å) column (Phenomenex, Le Pecq, France) using a linear gradient (50–90% over 45 min) of acetonitrile/TFA (99.9:0.1) at a flow rate of 10 mL/min. The purified peptides were then characterized by MALDI-TOF mass spectrometry on an ultrafleXtreme (Bruker, Strasbourg, France) in the reflector mode using α-cyano-4-hydroxycinnamic acid as a matrix. Analytical RP-HPLC, performed on a 4.6 × 250 mm Jupiter C18 (5 µm, 300 Å) column, indicated that the purity of all peptides was >99.9%. The net peptide content of lyophilized and dry peptide samples is assessed by CHN elemental analysis (N-content) using an Unicube elemental analyzer (Elementar, Lyon, France). Briefly, samples were accurately weighed (∿ 200 µg) into tin boats and compressed with a manual pressing tool in order to remove remaining air from the packed sample and then combusted in pure oxygen under static conditions resulting in the formation of CO_2_, NOx, H_2_ and H_2_O. The NOx compounds were reduced to N_2_ using elemental cupper. Gases were separated by gas chromatography and detected quantitatively (acetanilide as calibrant) by a thermal conductivity detector.

Listeriolysin O (LLO) was purified from *Escherichia coli* BL21(DE3)pLysS (Invitrogen) transformed with a plasmid coding for a hexahistidine tagged, signal peptide deficient version of the *L. monocytogenes* LLO gene, as previously described.^19^

### Co-incubation of cells with bacterial supernatants and toxins

The day before co-incubations, Caco2, HeLa and HIEC-6 cells were seeded at a density of 1.0 × 10^5^ cells/cm^2^ and RAW264.7 cells were seeded at a density of 2.0 × 10^5^ cells/cm^2^. *S. warneri* and *S. epidermidis* were cultured overnight at 37°C. Before co-incubations, cells were incubated in FBS-, antibiotics- and EGF-free culture medium. Where indicated, cells were pretreated for 2 h with 10 µM MG132 (M7449, Sigma-Aldrich) or incubated in MEM supplemented with 135 mM potassium chloride (KCl). Bacterial cultures were diluted in BHI to obtain a suspension with an optical density (OD) at 600 nm of 13.0. Supernatants were then collected after centrifugation for 2 min at 19,000 × *g* at room temperature. Supernatants were filtered (0.2 µm filter, Sartorius), treated or not for 1 h at 56°C with 0.4 mg/mL proteinase K (P8107S, New England Biolabs) and then heated or not for 1 h at 85°C.

Supernatants and purified toxins were directly added to the cell culture medium, as indicated in the text. After incubations, cells were washed twice with 1X PBS and lysed directly either in Laemmli buffer (4% SDS, 20% glycerol, 125 mM Tris-HCl [pH 6.8], 100 mM dithiothreitol [DTT], 0.02% bromophenol blue), for immunoblot analyses, or in RLT buffer (Qiagen), for RNA extraction.

### Immunoblot analysis

Cells lysates in a Laemmli buffer were boiled for 5 min, sonicated and their protein contents were analyzed by electrophoresis on TGX Stain-free pre-cast SDS-polyacrylamide gel (Bio-Rad). Proteins were then transferred to PVDF membranes (GE Healthcare) and detected after incubation, with specific antibodies using ECL Clarity Western blotting Substrate (Bio-Rad). Mouse anti-actin (A5441, Sigma-Aldrich, 1:10,000 dilution), mouse anti-Ubc9 (610748, BD Biosciences, 1:1,000 dilution), rabbit anti-SAE1 (#13585, Cell Signaling Technology, 1:1,000 dilution or 10,229–1-AP, Proteintech, 1:1,000 dilution), rabbit anti-SAE2 (D15C11, Cell Signaling Technology, 1:1,000 dilution), rabbit anti-SUMO3 (R205 (is2),^28^ 1:5,000 dilution) and rabbit anti-K48-linkage specific polyubiquitin (D9D5, Cell Signaling Technology, 1:1,000 dilution) antibodies were used as primary antibodies for immunoblotting experiments. HRP-conjugated goat anti-rabbit IgG (H+L) (BI 2407, Abliance, 1:5,000 dilution) and anti-mouse IgG (H+L) (BI 2413C, Abliance, 1:5,000 dilution) antibodies were used as secondary antibodies for immunoblotting experiments. Quantifications of proteins were performed on a ChemiDoc Imaging System (Bio-rad). Quantities of SUMO-conjugated proteins (above 50 kDa), polyubiquitylated proteins (K48-linkage; above 50 kDa), SAE1, SAE2 and Ubc9 were normalized by actin levels.

### Quantification of proinflammatory cytokines expression

Where indicated, cells incubated in FBS- and antibiotics-free culture medium were pre-treated with 10 µM TAK981 (Subasumstat, MedChemExpress) for 2 h or with Warnericin RK (20 µM for Caco2 cells and 10 µM for RAW264.7 cells) for 1 h. 1 µg/mL Pam_3_CSK_4_ (InvivoGen), 100 ng/mL recombinant human TNFα (PeproTech) or 50 ng/mL LPS (ultrapure LPS from *E. coli* 0111:B4; InvivoGen) were then added directly to the cell culture medium. After four additional hours of incubation, cells were washed twice with 1× PBS, and total RNAs were extracted using the RNeasy Plus Mini kit (Qiagen). About 0.5 to 1 µg of total RNAs were reverse transcribed using random hexamers and M-MLV reverse transcriptase (Invitrogen). Quantification of gene expression was performed by qPCR using Itaq Universal SYBR Green Supermix (Bio-Rad). GAPDH was used as an internal reference for normalization. Primers used in this study are listed in Table S1. Serial dilutions of target cDNAs were included on each plate to generate a relative curve and to integrate primer efficiency in the calculations of gene expression.

### Quantification of hemolytic activity

Hemolytic activities of *S. warneri* supernatant, Warnericin RK and delta-lysin II were determined by detecting the release of hemoglobin from sheep erythrocytes, as previously described.^29^ Briefly, sheep erythrocytes washed in 1× PBS were incubated with purified toxins or dilutions of *S. warneri* supernatants for 30 min at 37°C and then centrifugated 5 min at 2,000 × *g* to pellet unlysed red blood cells. The OD at 540 nm of the resulting supernatants was then measured to estimate the percentage of lysed sheep erythrocytes. Erythrocytes hypotonically lysed in distilled water were used as positive controls (100% hemolysis).

### Statistical analysis

Results are expressed as mean ± standard deviation (s.d.). Statistical analyses were performed with GraphPad Prism 9.5 (GraphPad Software Inc., San Diego, CA, USA). As indicated in the corresponding figure legends, two-tailed Student’s t-test was used to compare two groups, whereas one-way ANOVA with Dunnett’s, Tukey's or Sidak’s correction was used to compare more than two groups. Means with *p*-values below 0.05 (*p* < 0.05) were considered significantly different.

## Results

### S. warneri *inhibits host cell SUMOylation*

**Figure 1.**
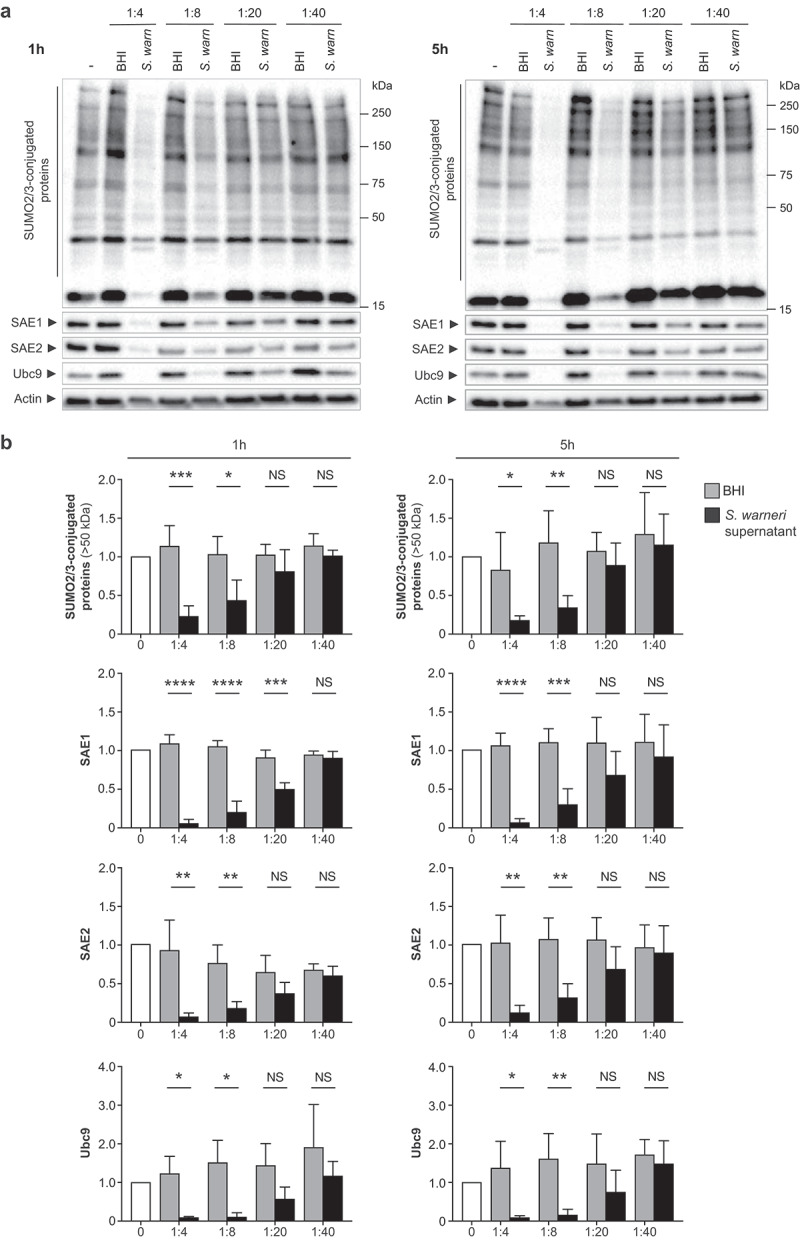
*Staphylococcus warneri* decreases SUMOylation in HeLa cells. (a) immunoblot analysis of SUMO2/3-conjugated proteins, SAE1, SAE2 and Ubc9 in HeLa cells treated with dilutions of *S. warneri* supernatant (*S. warn*) or with bacterial culture medium alone (BHI) for 1 h (left) or 5 h (right). (b) quantification of SUMO2/3-conjugated proteins (above 50 kDa), SAE1, SAE2 and Ubc9 levels after normalization by actin levels. Values are expressed as fold-change versus untreated cells (mean ± s.d.; *n* = 3–4; *, *P*<0.05; **, *P*<0.01; ***, *P*<0.001; ****, *P*<0.0001; NS, not significant; two-tailed Student’s t-test).

To assess whether *S. warneri* regulates host cell SUMOylation, we first compared the global pattern of SUMO2/3-conjugated proteins in a human cell line (HeLa cells) incubated or not with *S. warneri* supernatant. Of note, HeLa cells, which are a cervical cancer cell line, were used here as a model of human epithelial cells (cervical cells are not expected to be exposed to *S. warneri in vivo*). We observed that *S. warneri* supernatant induces a significant decrease in the level of SUMO-conjugated proteins after 1 h or 5 h of incubation (Figure 1a,b). This decrease in SUMOylation is dependent on the amount of *S. warneri* supernatant added to cells (Figure 1).

As several host targets are deSUMOylated in response to *S. warneri* supernatant, we hypothesized that this bacterium directly impacts the host SUMOylation machinery, as already observed for pathogenic bacteria such as *L. monocytogenes* .^19^ We thus quantified the levels of E1 and E2 enzymes in HeLa cells incubated with *S. warneri* supernatant and observed a significant decrease in both Ubc9, SAE1 and SAE2 levels, in a dilution-dependent manner (Figure 1a,b). Since SUMOylation levels correspond to a dynamic equilibrium between SUMO-conjugation and deSUMOylation reactions, this decrease in the level of SUMO-conjugating enzymes is likely responsible for the global decrease in SUMOylation triggered by *S. warneri*.

Together, these results suggest that *S. warneri* dampens host SUMOylation by secreting an effector targeting the human SUMO conjugation machinery.

**Figure 2.**
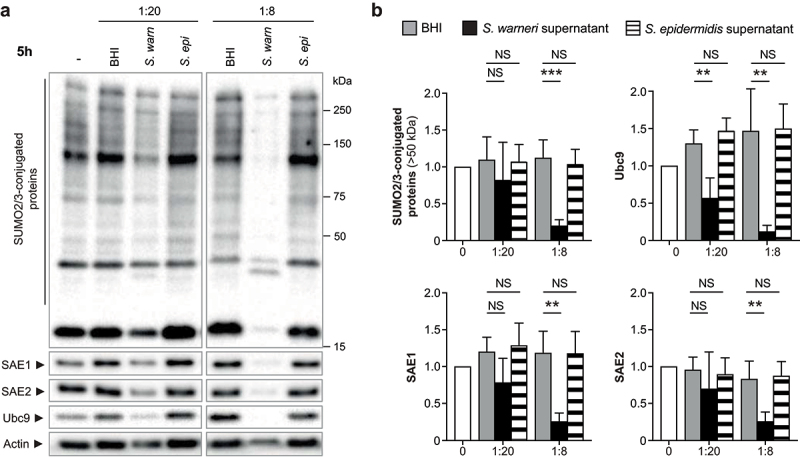
*S. epidermidis* does not inhibit host cell SUMOylation. (a) immunoblot analysis of SUMO2/3-conjugated proteins, SAE1, SAE2 and Ubc9 in HeLa cells treated with dilutions of *S. warneri* (*S. warn*) or *S. epidermidis* (*S. epi*) supernatants, or with bacterial culture medium alone (BHI), for 5 h. (b) quantification of SUMO2/3-conjugated proteins (above 50 kDa), SAE1, SAE2 and Ubc9 levels after normalization by actin levels. Values are expressed as fold-change versus untreated cells (mean ± s.d.; *n* = 4; **, *P*<0.01; ***, *P*<0.001; NS, not significant; one-way ANOVA, with Dunnett’s correction).

To determine whether the alteration of host SUMOylation triggered by *S. warneri* is specific to this bacterial species, we performed similar experiments with the supernatant of *Staphylococcus epidermidis*, another CoNS species. Interestingly, in contrast to *S. warneri*, we did not observe any changes in the global SUMOylation pattern of HeLa cells incubated with high (1:20) and low (1:8) dilutions of *S. epidermidis* supernatant for 5 h (Figure 2a). The level of SUMO E1 and E2 enzymes remained unchanged in response to *S. epidermidis* (Figure 2b). These data suggest that the effector secreted by *S. warneri* and targeting host SUMOylation is absent from the supernatant of *S. epidermidis*.

### *Identification of the* S. warneri *effector triggering host protein deSUMOylation*

**Figure 3.**
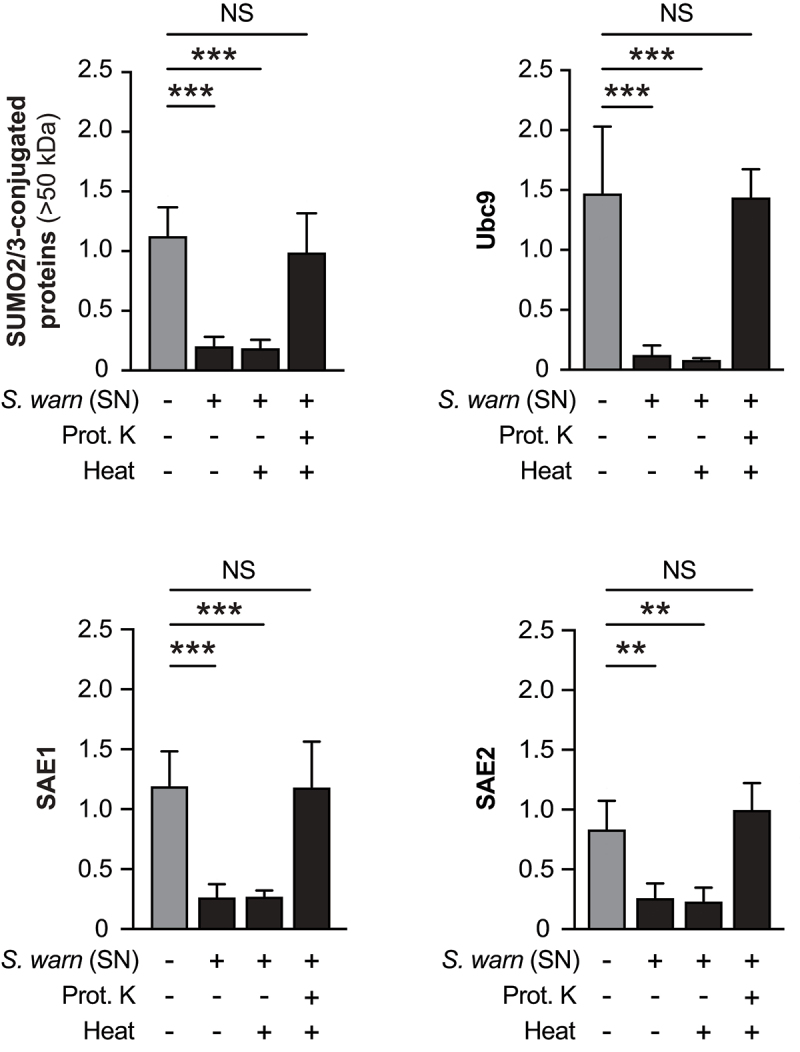
Characterization of the *S. warneri* effector interfering with SUMOylation. Quantification of SUMO2/3-conjugated proteins (above 50 kDa), SAE1, SAE2 and Ubc9 levels, after normalization by actin levels, in HeLa cells treated for 5h with bacterial culture medium alone (BHI; grey bars) or with *S. warneri* supernatant (SN) (1:8 dilution), with or without heat or proteinase K (prot. K) pre-treatments . Values are expressed as fold-change versus untreated cells (mean ± s.d.; *n* = 4; **, *P*<0.01; ***, *P*<0.001; NS, not significant; one-way ANOVA, with Dunnett’s correction).

In order to characterize the effector secreted by *S. warneri* responsible for the observed decrease in host protein SUMOylation, we pre-treated *S. warneri* supernatant with proteinase K, a broad-spectrum serine protease. We also tested the effect of heat inactivation on *S. warneri* supernatant. HeLa cells were then incubated with these supernatants for 5 h. We observed that heat inactivation did not impair *S. warneri*-induced deSUMOylations (Figure 3). In contrast, pre-treatment with proteinase K induces a complete blocking of *S. warneri*-induced deSUMOylations, as well as a lack of decrease in SUMO E1 and E2 levels (Figure 3). Together, these results suggest that the effector secreted by *S. warneri* and involved in host protein deSUMOylation is a heat-resistant protein or peptide.

**Figure 4.**
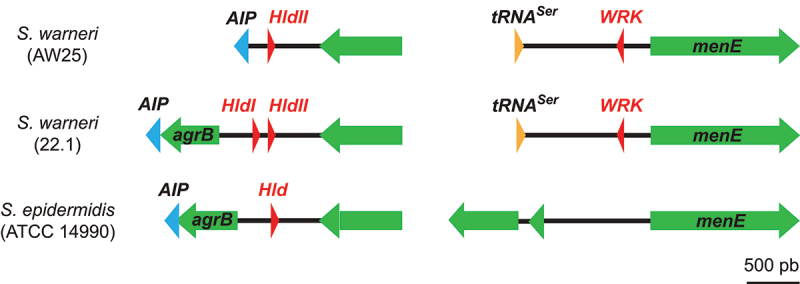
Genetic loci of hemolytic peptides encoded by *S. warneri* and *S.* *epidermidis. Schematic representation of the genetic loci coding for* W*arnericin RK (WRK) and delta-lysins (Hld) in S.* *warneri AW25 type strain, S.* *warneri 22.1 strain and in S.* *epidermidis ATCC 14990 type strain.*

Since the effect of *S. warneri* on host SUMOylation is similar to the one described for *L. monocytogenes* or other bacterial pathogens secreting pore-forming toxins,^17,19^ we focused on potential pore-forming proteins or peptides produced by *S. warneri*, which would not be encoded by *S. epidermidis*. Three peptides, named Warnericin RK (WRK), delta-lysin I (HldI) and delta-lysin II (HldII), were identified in various strains of *S. warneri* and were reported to exhibit hemolytic activities.^30,31^ HldI and HldII belong to a family of peptides called delta-hemolysins (Hld), which are also encoded by *S. epidermidis* and other Staphylococcal species.^31^ In contrast, WRK has no homolog in *S. epidermidis* (Figure 4).^30^ We thus tested the potential effect of WRK on host cell SUMOylation. We also tested, as a control, the effect of the HldII peptide (the gene coding for HldI being deleted in the *S. warneri* AW25 strain used in our experiments).

**Figure 5.**
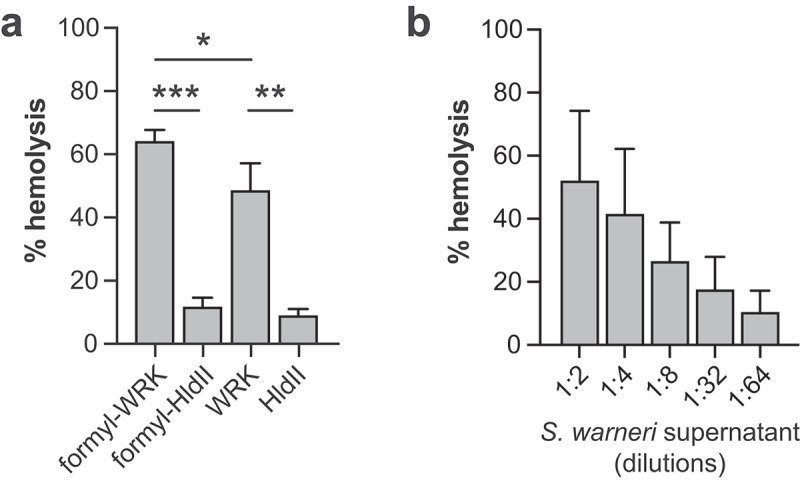
Hemolytic activities of Warnericin RK and delta-lysin II. (a) hemolytic activities of 25 µM WRK, formyl-WRK, HldII and formyl-HldII. Values are expressed as percentage of red blood cell lysis compared to a positive reference (100% lysis) (mean ± s.d.; *n* = 3; *, *P*<0.05, **; *P*<0.01; ***, *P*<0.001; one-way ANOVA, with Tukey's correction). (b) hemolytic activity of *S. warneri* supernatant (mean ± s.d.; *n* = 3).

Both Warnericin RK and HldII peptides were synthetized, either in their unmodified form or with an N-terminal formylation (formyl-WRK and formyl-HldII). Indeed, formylation of the N-terminal methionine residue has been reported for both peptides.^30^ We first performed hemolytic assays to confirm the ability of these peptides to destabilize eukaryotic plasma membranes. We observed that both WRK and HldII peptides exhibit hemolytic activity and that the activity of WRK is significantly higher than the one of HldII (Figure 5a). Of note, N-formylation of WRK increases its hemolytic activity. We confirmed in parallel that the supernatant of *S. warneri* grown in the stationary phase also exhibits hemolytic activity (Figure 5b).

**Figure 6.**
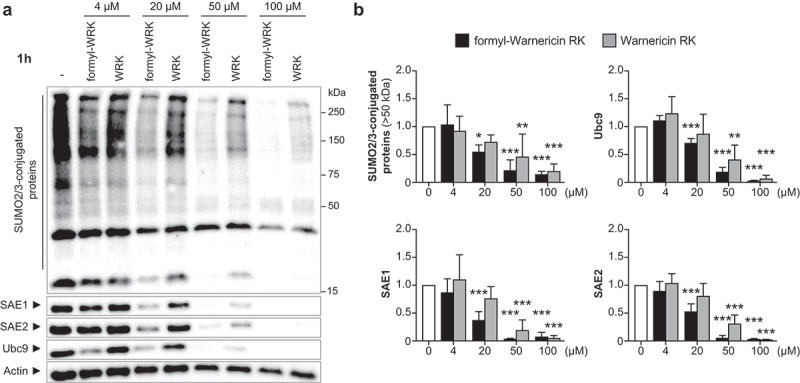
Warnericin RK decreases protein SUMOylation in HeLa cells. (a) immunoblot analysis of SUMO2/3-conjugated proteins, SAE1, SAE2 and Ubc9 in HeLa cells treated or not with formyl-WRK or WRK for 1 h. (b) quantification of SUMO2/3-conjugated proteins (above 50 kDa), SAE1, SAE2 and Ubc9 levels, after normalization by actin levels. Values are expressed as fold-change versus untreated cells (mean ± s.d.; *n* = 4–5; *, *P*<0.05; **, *P*<0.01; ***, *P*<0.001; one-way ANOVA, with Dunnett’s correction).

We then investigated the effect of Warnericin RK and delta-lysin II on human cell SUMOylation. For this, we co-incubated HeLa cells with various concentrations of WRK and HldII peptides for 1 h and analyzed the level of SUMO E1 and E2 enzymes as well as the global pattern of SUMO2/3-conjugated proteins by immunoblotting experiments. Strikingly, we observed that WRK is sufficient to decrease the level of both SUMO E1 and E2 enzymes and to trigger a global dampening of host protein SUMOylation in a dose-dependent manner (Figure 6). Of note, the effect of the formylated form of WRK on host SUMOylation machinery is slightly stronger than the N-terminal-free form (Figure 6). We performed in parallel viability assays and observed a limited toxicity of WRK at 20 µM on HeLa cells after 1 h of incubation (<30%) (Figure S1A). In contrast to WRK, the HldII peptide does not significantly affect host SUMOylation, even at high concentrations (100 µM) (Figure S2). Together, these results demonstrate that Warnericin RK targets the host SUMOylation machinery and triggers a deSUMOylation of host proteins.

### Warnericin RK affects intestinal SUMOylation

**Figure 7.**
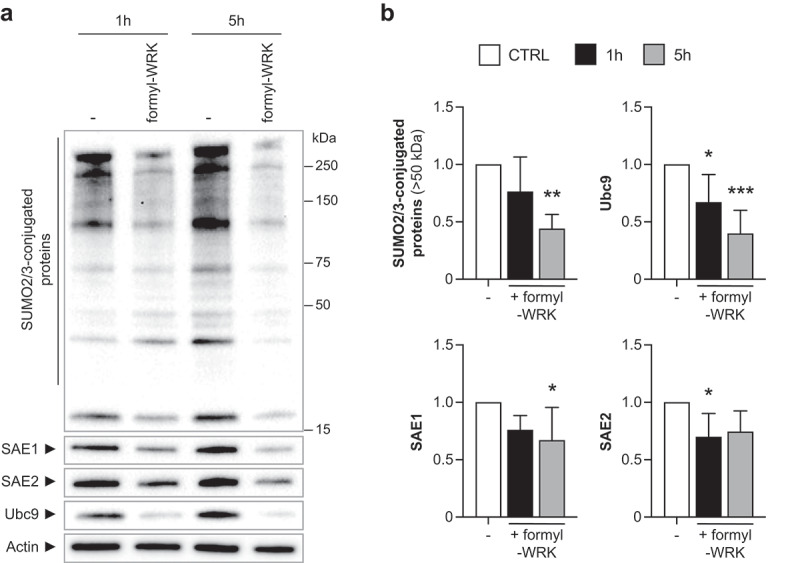
Warnericin RK induces deSUMOylation in intestinal cells. (a) immunoblot analysis of SUMO2/3-conjugated proteins, SAE1, SAE2 and Ubc9 in Caco2 cells treated with 20 µM formyl-WRK for 1 h and 5 h. (b) quantification of SUMO2/3-conjugated proteins (above 50 kDa), SAE1, SAE2 and Ubc9 levels, after normalization by actin levels. Values are expressed as fold-change versus untreated cells (mean ± s.d.; *n* = 4–5; *, *P*<0.05; **, *P*<0.01; ***, *P*<0.001; one-way ANOVA, with Dunnett’s correction).

As *S. warneri* is a natural member of the human gut microbiota, we tested the effect of WRK on the SUMOylation of intestinal cells, which are exposed *in vivo* to this bacterium. For this, we first used Caco2 cells that we incubated with various concentrations of formyl-WRK. We observed that exposition of Caco2 cells to concentrations of formyl-WRK above 20 µM is associated with a significant toxicity (Fig. S1B). We thus used 20 µM formyl-WRK to assess the impact of this toxin on Caco2 SUMOylation. Caco2 cells were incubated for 1 and 5 h with 20 µM formyl-WRK and the level of SUMO E1 and E2 enzymes, as well as the global pattern of SUMO2/3-conjugated proteins, were then analyzed by immunoblotting experiments (Figure 7). After 5 h of incubation, we observed a significant decrease in the overall SUMOylation profile, as well as a decrease in the level of Ubc9, as previously observed in HeLa cells (Figure 7).

To complete these results, we performed similar analyses in HIEC6, a non-immortalized, continuously growing human intestinal cell line expressing crypt features.^32^ We observed that these cells were highly sensitive to WRK since a significant decrease in cell viability was observed for WRK concentrations above 5 µM and an alteration of cell morphology observed for concentrations as low as 2.5 µM (Figure S3). We thus used low levels of formyl-WRK (≤5 µM) to assess the potential impact of this toxin on HIEC6 SUMOylation. We did not observe any impact of WRK on SUMOylation for these concentrations of WRK (Figure S3).

Together, these results indicate that Warnericin RK promotes protein deSUMOylation in different human cell types, including some intestinal cells.

### Characterization of the mechanisms of Ubc9 degradation triggered by Warnericin RK

**Figure 8.**
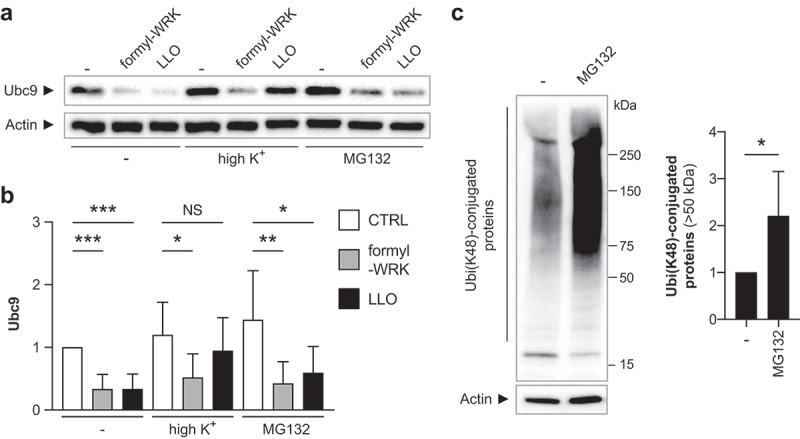
Warnericin RK-mediated degradation of Ubc9 is potassium efflux- and proteasome-independent. (a) immunoblot analysis of Ubc9 in Caco2 cells pre-treated or not with high extracellular concentration of potassium chloride (high K^+^), or with MG132 for 2 h, and then treated or not with 20 µM formyl-WRK for 1 h or 3 nM LLO for 30 minutes. (b) quantification of Ubc9 levels, after normalization by actin levels. Values are expressed as fold-change versus untreated cells (mean ± s.d.; *n* = 6–9; *, *P*<0.05; **, *P*<0.01; ***, *P*<0.001; NS, not significant; one-way ANOVA, with Dunnett’s correction). (c) immunoblot analysis and quantification of Ubi-conjugated proteins (K48-linked chains) in Caco2 cells pre-treated or not with MG132 for 2 h. Values are expressed as fold-change versus untreated cells (mean ± s.d.; *n* = 6; *, *P*<0.05; two-tailed Student’s t-test).

The impact of WRK on protein SUMOylation is similar to that triggered by Listeriolysin O.^19^ This toxin is indeed able to induce the degradation of the SUMO E2 enzyme in human cells and to decrease host SUMOylation.^19,20^ It has been shown that LLO-mediated degradation of Ubc9 is initiated by the efflux of potassium generated by the formation of pores by LLO in host cell plasma membranes.^33,34^ Since we showed that Warnericin RK is also able to permeabilize host membranes (Figure 5), we investigated whether potassium efflux also initiates SUMO E2 enzyme degradation in response to Warnericin RK. To do so, we incubated Caco2 cells with an excess of extracellular KCl to block potassium efflux from the intracellular compartment following plasma membrane permeabilization. Caco2 cells were then incubated with 20 µM formyl-WRK for 1 h or with 3 nM LLO for 30 min (Figure 8). As expected, we observed that LLO induced a significant decrease in Ubc9 levels that was blocked by an excess of extracellular KCl. In contrast to LLO, inhibiting potassium effluxes has no impact on the degradation of Ubc9 triggered by formyl-WRK (Figure 8a,b). These results suggest that degradation of the SUMO E2 enzyme by formyl-WRK does not depend on potassium efflux and thus uses a different pathway than the one triggered by LLO.

To complete these results, we also determined whether Ubc9 degradation was proteasome-dependent. For this, we treated Caco2 cells with the proteasome inhibitor MG132 for 2 h and then incubated cells with 20 µM formyl-WRK for 1 h or 3 nM LLO for 30 min (Figure 8a,b). Proteasome inhibition by MG132 was confirmed by the observation of an accumulation of host proteins conjugated to K48 linked-ubiquitin chains in Caco2 cells (Figure 8c). We observed that both formyl-WRK- and LLO-mediated Ubc9 degradation are not inhibited by MG132, indicating that these degradation mechanisms are proteasome-independent (Figure 8a,b).

### Warnericin RK alters gene expression in intestinal cells

As SUMOylation is known to regulate inflammation,^13,14^ we investigated whether WRK-induced deSUMOylations modulate intestinal cells inflammatory responses. For this, Caco2 cells were first pre-treated or not with formyl-WRK for 1 h and then incubated for 4 h with recombinant human TNFα or Pam_3_CSK_4_, a synthetic triacylated lipopeptide acting as a ligand of Toll-like Receptor 1/2 (TLR1/2). After incubation, the expression levels of IL8, IL23A, S100A9 and PYCARD genes were quantified by qRT-PCR. As a control, we treated cells with TAK981, a selective inhibitor of SUMOylation targeting the SUMO E1 enzymes,^35^ to mimic WRK-induced deSUMOylations.

**Figure 9.**
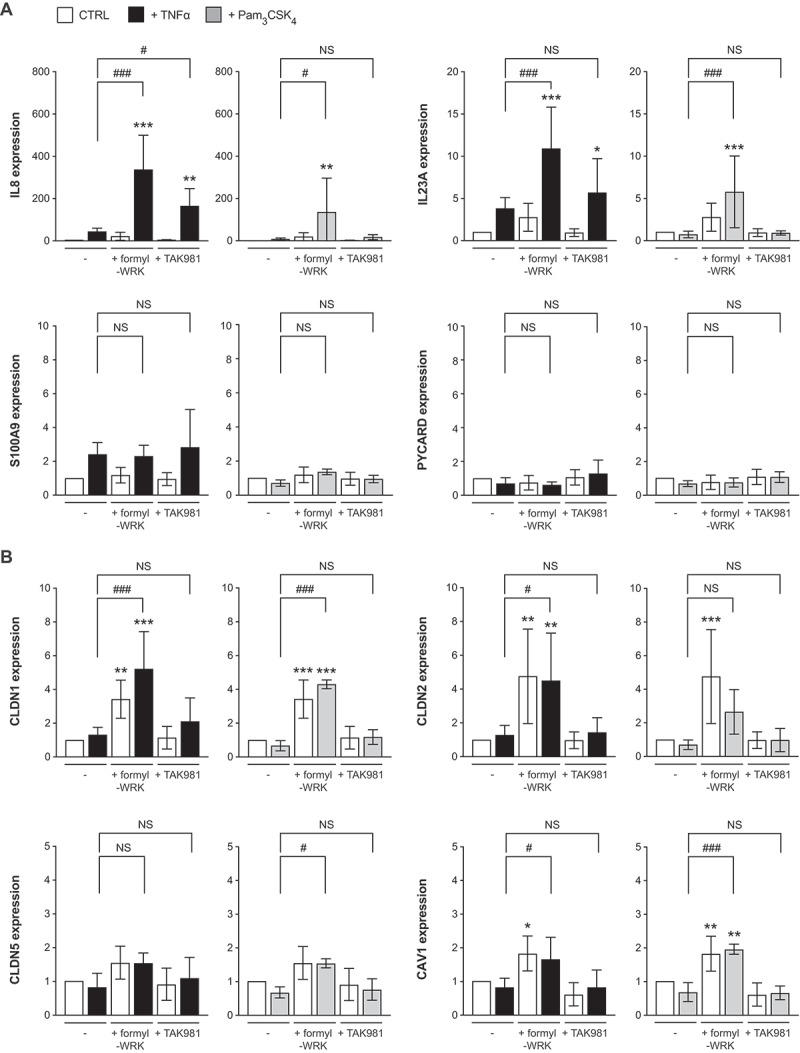
Warnericin RK regulates the expression of genes involved in inflammatory responses and tight junction formation in Caco2 cells. Expression level of genes involved in inflammatory responses (a) or tight junction formation (b) in Caco2 cells pre-treated or not with formyl-WRK for 1 h, or TAK981 for 2 h, and then incubated for 4 h with 100 ng/mL TNFα or 1 µg/mL Pam_3_CSK_4_. Values are expressed as fold change versus untreated cells (mean ± s.d.; *n* = 3–6; NS, not significant; *, *P*<0.05; **, *P*<0.01; ***, *P*<0.001 versus untreated cells; #, *P*<0.05; ##, *P*<0.01; ###, *P*<0.001 versus cells treated with TNFα or Pam_3_CSK_4_; one-way ANOVA, with Sidak’s correction).

Interestingly, we observed that pre-treatment with formyl-WRK strongly enhances IL8 expression in response to TNFα (Figure 9a). Similar effects on IL8 were observed in cells pre-treated with TAK981, thus suggesting that the effect of WRK on IL8 expression is due to the deSUMOylations triggered by this bacterial toxin. For IL23A, we also observed an enhanced expression in response to TNFα with formyl-WRK but not with TAK981. This suggests that formyl-WRK regulates IL23A gene expression using SUMO-independent mechanisms. These SUMO-independent regulations are also observed for IL8 and IL23A gene expression in response to Pam_3_CSK_4_ (Figure 9a). This enhancement is not observed for other genes involved in inflammatory responses such as S100A9 or PYCARD. Together, these results demonstrate that formyl-Warnericin RK enhances TNFα- and Pam_3_CSK_4_-induced inflammatory responses in intestinal cells using both SUMO-dependent and SUMO-independent mechanisms.

In addition to genes involved in inflammatory responses, we monitored the expression level of genes involved in tight junction formation and intestinal permeability. We observed that formyl-WRK alone, *i.e*. without incubation with TNFα nor Pam_3_CSK_4_, significantly increases the expression of claudin-1 (CLDN1), claudin-2 (CLDN2) and caveolin-1 (CAV1). This effect was not observed for claudin-5 (CLDN5) (Figure 9b). The enhancement of claudin-1, claudin-2 and caveolin-1 expression triggered by formyl-WRK is SUMO-independent since no modification was observed in response to TAK981 pre-incubation. Together, these results indicate that formyl-WRK, in addition to its effect on inflammatory responses, also remodels the expression of genes involved in tight junction formation and may impact intestinal permeability.

### Warnericin RK inhibits SUMOylation in immune cells and promotes inflammatory responses

**Figure 10.**
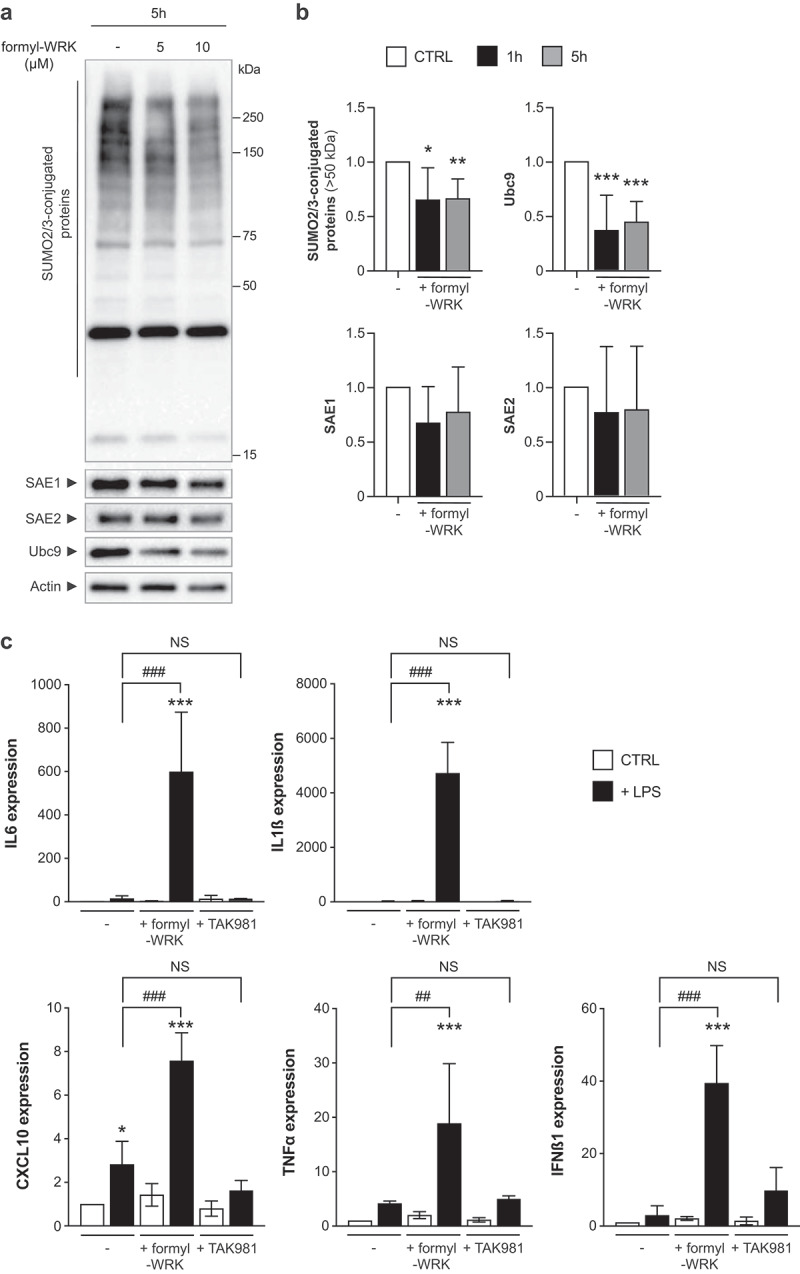
Warnericin RK induces deSUMOylation and enhances inflammatory responses in macrophages. (a) immunoblot analysis of SUMO2/3-conjugated proteins, SAE1, SAE2 and Ubc9 in RAW264.7 cells treated with 5 or 10 µM formyl-WRK for 5 h. (b) quantification of SUMO2/3-conjugated proteins (above 50 kDa), SAE1, SAE2 and Ubc9 levels in RAW264.7 cells treated with 10 µM formyl-WRK for 1 h or 5 h, after normalization by actin levels. Values are expressed as fold-change versus untreated cells (mean ± s.d.; *n* = 5-8; *, *P*<0.05; **, *P*<0.01; ***, *P*<0.001; NS, not significant; one-way ANOVA, with Dunnett’s correction). (c) expression level of genes involved in inflammatory responses in RAW264.7 cells pre-treated or not with formyl-WRK for 1 h, or TAK981 for 2 h, and then incubated for 4 h with 50 ng/mL LPS. Values are expressed as fold change versus untreated cells (mean ± s.d.; *n* = 4; NS, not significant; *, *P*<0.05; ***, *P*<0.001 versus untreated cells; ##, *P*<0.01; ###, *P*<0.001 versus cells treated with LPS; one-way ANOVA, with Sidak’s correction).

In order to determine whether Warnericin RK also affects immune cells, we investigated the impact of this toxin on the SUMOylation of the macrophage cell-line RAW264.7. Of note, RAW264.7 cells are more sensitive to WRK than HeLa and Caco2 cells since significant toxicity was observed for concentration above 10 µM after 5 h (Figure S1C). To test the effect of WRK on RAW264.7 SUMOylation, we incubated cells with a low concentration of formyl-WRK (5 or 10 µM) for 1 or 5 h. The global pattern of SUMO2/3-conjugated proteins and the level of SUMO E1 and E2 enzymes were analyzed by immunoblotting. We observed a significant decrease in the overall SUMOylation pattern, as well as a decrease in the level of Ubc9 (Figure 10a,b), as previously observed for HeLa and Caco2 cells. In contrast, no significant modification in the level of SAE1 or SAE2 was observed (Figure 10a,b). These results demonstrate that Warnericin RK also induces deSUMOylations in immune cells, by targeting the SUMO E2 enzyme.

We then investigated whether WRK-induced deSUMOylations modulate inflammatory responses in macrophages. For this, RAW264.7 cells were first pre-treated or not with formyl-WRK for 1 h and then incubated for 4 h with LPS. After incubation, the expression levels of CXCL10, IFNß1, IL1ß, IL6 and TNFα genes were quantified by qRT-PCR. As a control, we treated cells with TAK981 to mimic WRK-induced deSUMOylations. We observed that formyl-WRK strongly enhances the expression of all the tested genes in the presence of LPS (Figure 10c). This enhancement was not observed with TAK981, which suggests that these genes are regulated by formyl-WRK in a SUMO-independent manner (Figure 10c).

Together, these results demonstrate that formyl-WRK has pleiotropic effects on immune cells as it both decreases SUMOylation and promotes the expression of genes involved in inflammation in a SUMO-independent manner.

## Discussion

SUMOylation is a post-translational modification playing key roles in the maintenance of intestinal epithelium integrity and in the regulation of inflammatory responses.^4,12,14^ Several gut bacterial pathogens were shown to interfere with host intestinal SUMOylation.^17,18^ These pathogens use various mechanisms to dampen SUMO conjugation. Most of them directly target the SUMOylation machinery (either the SUMO E1, E2 or E3 enzymes) but bacterial virulence effectors with deSUMOylase activities have also been reported.^17,18^ In contrast to pathogenic bacteria, mutualistic bacteria from the gut microbiota promote intestinal SUMOylation, via, for example, the production of SCFAs/BCFAs.^4^ These bacterial metabolites, which are abundant in the gut, inactivate intestinal deSUMOylases and promote hyperSUMOylation of nuclear proteins.^4^ Here, we show that *S. warneri*, a bacterium from the human gut microbiota, has a similar impact on host SUMOylation than pathogens, as it dampens intestinal SUMOylation. *S. warneri*-induced decrease in SUMO-conjugated proteins is associated with a decrease in the level of SUMO E1 and E2 enzymes. As described above, the SUMOylation level of proteins results from the dynamic balance between SUMO-conjugation and SUMO-deconjugation reactions. Inhibition of SUMO-conjugation reactions shifts this equilibrium toward the deSUMOylated forms of proteins, due to the high activity of cellular deSUMOylases. The set of SUMO-conjugated proteins affected by this process would thus depend on their sensitivity/accessibility to deSUMOylases.

We identified Warnericin RK as the bacterial effector encoded by *S. warneri* responsible for this decrease in SUMO-conjugated proteins. This 22 amino acids-long peptide was initially identified for its antimicrobial activity against *Legionella pneumophila*.^30^ It has been proposed that Warnericin RK kills *Legionella* by acting as a detergent on bacterial membranes. We show that, in addition to its antimicrobial activity, Warnericin RK also acts on eukaryotic cells. This versatility of activities has been observed in many other examples of antimicrobial peptides.^36^

The mechanism by which Warnericin RK forms pores or destabilizes eukaryotic membranes remains to be characterized. We show that formylated Warnericin RK has a higher hemolytic activity than N-terminal-free Warnericin RK, as well as a more potent effect on host SUMOylation (Figure 5). It has been proposed that formylation blocks the N-terminal amine group of Warnericin RK which thus cannot be protonated, thereby decreasing its net positive charge and increasing its hydrophobicity.^37^ This increase in hydrophobicity probably facilitates the destabilization of eukaryotic membranes and the downstream induction of host deSUMOylation. Interestingly, we show that delta-hemolysin II, another hemolytic toxin produced by *S. warneri*, and also encoded by *S. epidermidis*, has no impact on host SUMOylation. This lack of effect might be due to the lower hemolytic activity of this toxin (Figure 5).

Interestingly, the effect of Warnericin RK on host SUMOylation is quite similar to that of the *L. monocytogenes* pore-forming toxin LLO. In the case of LLO, pores formed by the toxin after binding to host membranes generates an efflux of potassium that initiates a signaling cascade leading to the degradation of Ubc9 and to a decrease in SUMO-conjugated proteins.^19^ Here, we show that Warnericin RK mediates Ubc9 degradation using a potassium efflux-independent and proteasome-independent mechanism. This result illustrates an example of convergent evolution where two bacterial toxins use independent mechanisms to target a similar host factor. Proteins deSUMOylated in response to LLO were mainly shown to be transcription factors.^20^ Whether deSUMOylated targets are similar between Warnericin RK and LLO remains to be determined.

In addition to its impact on host SUMOylation, *S. warneri* shares other similarities with *bona fide* intestinal pathogens, since this bacterium is able to get internalized into human intestinal cells.^24^ Our work now paves the way for future studies to determine whether formyl-Warnericin RK is involved in *S. warneri* internalization, whether deSUMOylations triggered by *S. warneri* have an impact on the intracellular fate of the bacterium and whether it contributes to the consequences of *S. warneri* internalization on intestinal cell responses.

We show that Warnericin RK induces deSUMOylation in several independent cell lines, including epithelial cells and macrophages. In the case of intestinal cells, we observed an effect of Warnericin RK on Caco2 cells. We additionally assessed the effect of Warnericin RK on HIEC6 cells, a non-immortalized intestinal cell line. We did not observe an effect of WRK on HIEC6 SUMOylation. This may indicate that Warnericin RK targets SUMOylation only in specific intestinal cell types, although we cannot rule out that the sensitivity of HIEC6 to Warnericin RK was too high to detect any modification in SUMOylation. Our results indicate that the deSUMOylations triggered by Warnericin RK promote inflammatory responses in the intestinal epithelium. This toxin can indeed strongly enhance the expression of pro-inflammatory cytokines both in intestinal epithelial cells and in macrophages. These upregulations, triggered by Warnericin RK, use both SUMO-dependent and SUMO-independent mechanisms. Interestingly, previous studies showed that deSUMOylation of specific host proteins, such as PML, could be sensed as a danger signals by host cells and trigger anti-bacterial responses.^28^ More generally, since many transcription factors are regulated by SUMOylation, alteration of SUMO-conjugation may change the activity of these factors and thus participate in transcription remodeling.^38^ Besides its effect on inflammatory responses, we identified that Warnericin RK alters the expression of genes involved in tight junction formation such as claudin-1, claudin-2 or caveolin-1. Further experiments would now be required to determine whether these alterations are associated with a modification in intestinal permeability.

We identified here an example of gut bacteria dampening SUMOylation and promoting inflammation. This contrasts with the previously identified SCFA/BCFA-producing bacteria that increase SUMOylation and dampen inflammation. Our work thus unveils a balance in the gut between bacterial signals promoting or dampening SUMOylation. Changes in the composition of the gut microbiota, for example in the case of a dysbiosis, may thus alter this balance, with potential important repercussions on host intestinal physiology. Interestingly, deregulation of SUMOylation has been implicated in inflammatory diseases such as Inflammatory Bowel Diseases (IBD). Indeed, patients with Crohn’s disease or Ulcerative colitis show a decrease in colonic SUMOylation associated with a decrease in Ubc9 level.^13^ This decrease in Ubc9 level was also observed in a mouse model of colitis.^13^ By showing that deSUMOylation triggered by *S. warneri* promotes inflammatory responses, our results suggest that an increase in the abundance of *S. warneri* in the gut (or, more generally, an increase in bacteria inhibiting SUMOylation) may promote the onset or maintenance of inflammation and thus participate in intestinal inflammatory diseases. Quantifying the abundance of *S. warneri* in the gut microbiota of IBD patients, or monitoring the secretion of Warnericin RK in patients’ gut would help to validate this hypothesis.

In conclusion, our results unveil for the first time that gut bacteria such as *Staphylococcus warneri* modulate intestinal cell activity by dampening SUMOylation. This finding highlights the importance of SUMOylation in intestinal homeostasis and opens new research avenues to determine how disequilibrium between pro- and anti-SUMO gut bacterial effectors may lead to human intestinal pathologies.

## Supplementary Material

Supplemental Material

## Data Availability

The authors confirm that the data supporting the findings of this study are available within the article and its supplementary materials.
