# Supplementary material for: Staphylococcus warneri dampens SUMOylation and promotes intestinal inflammation
Source: Gut Microbes. 2025 Jan 16;17(1):2446392. doi: 10.1080/19490976.2024.2446392 (PMC12931719; doi:10.1080/19490976.2024.2446392)
Supplement: Supplemental Material [file KGMI_A_2446392_SM5982.pdf]

Target gene	Species	Forward primer	Reverse primer	T an[a]
CAV1	human	GCGACCCTAAACACCTCAAC	ATGCCGTCAAAACTGTGTGTC	60
CLDN1	human	CCAGTCAATGCCAGGTACGAAT	TTGGTGTTGGGTAAGAGGTTGTT	60
CLDN2	human	ATCGCTCCAACTACTACGATGC	TGAACTCACTCTTGACTTTGGGA	60
CLDN5	human	CTCTGCTGGTTCGCCAACAT	CAGCTCGTACTTCTGCGACA	60
GAPDH	human	TGCCATCAATGACCCCTTCA	TGACCTTGCCCACAGCCTTG	62
IL1ß	human	ACGAATCTCCGACCACCA	ATAAGCCTCGTTATCCCATG	60
IL8	human	TGGCAGCCTTCCTGATTT	AACTTCTCCACAACCCTCTG	60
IL23A	human	CTCAGGGACAACAGTCAGTTC	ACAGGGCTATCAGGGAGCA	60
PYCARD	human	TGGATGCTCTGTACGGGAAG	CCAGGCTGGTGTGAAACTGAA	60
S100A9	human	GCACCCAGACACCCTGAAC	GCTGCTTGTCTGCATTTGTGT	60
CXCL10	mouse	AATGAGGGCCATAGGGAAGC	ATCGTGGCAATGATCTCAACAC	57
GAPDH	mouse	CATCACTGCCACCCAGAAGA	AAGTCGCAGGAGACAACCT	60
IFNß1	mouse	TCCAGCTCCAAGAAAGGACG	TTGAAGTCCGCCCTGTAGGT	57
IL1ß	mouse	CCCAAAAGATGAAGGGCTGC	AAGGTCCACGGGAAAGACAC	64
IL6	mouse	CACTTCACAAGTCGGAGGCT	CTGCAAGTGCATCATCGTTGT	62
TNFα	mouse	GATCGGTCCCCAAAGGGATG	GGTTTGCTACGACGTGGGC	60

Table S1: Primer sequences

[a]Temperature of annealing

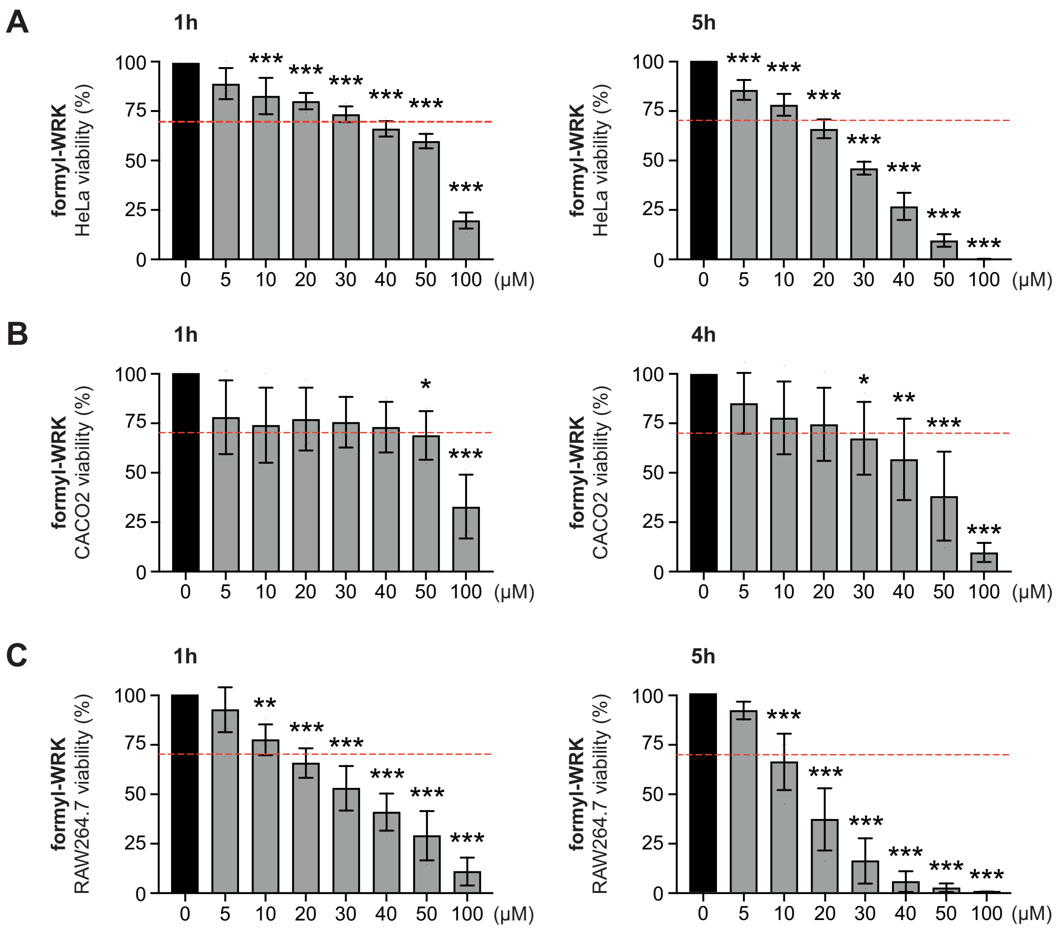

Figure S1: Viability of HeLa, Caco2 and RAW264.7 cells incubated with formyl-WRK. Viability of HeLa (A), Caco2 (B) and RAW264.7 (C) cells incubated for 1h, 4h or 5h with increasing concentrations of formyl-WRK (mean ± s.d.; n=4; *, $P<0.05$; **, $P<0.01$; ***, $P<0.001$; One-way ANOVA, with Dunnett's correction).

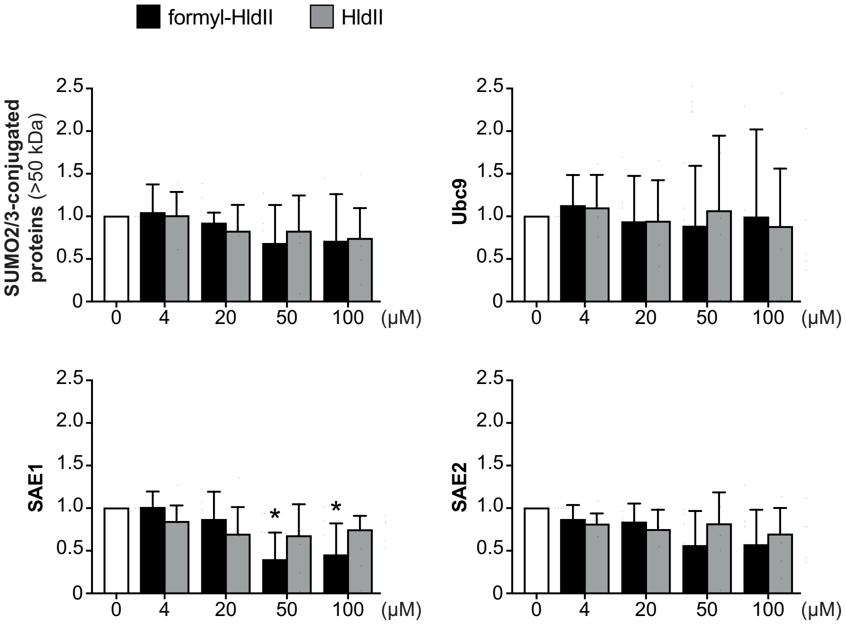

Figure S2: Lack of effect of delta-lysin II on SUMOylation in HeLa cells.

Quantification of SUMO2/3-conjugated proteins (above 50 kDa), SAE1, SAE2 and Ubc9 levels, after normalization by actin levels, in HeLa cells treated with HldII or formyl-HldII for 1h. Values are expressed as fold-change versus untreated cells (mean ± s.d.; n=4-5; *, $P<0.05$; One-way ANOVA, with Dunnett's correction).

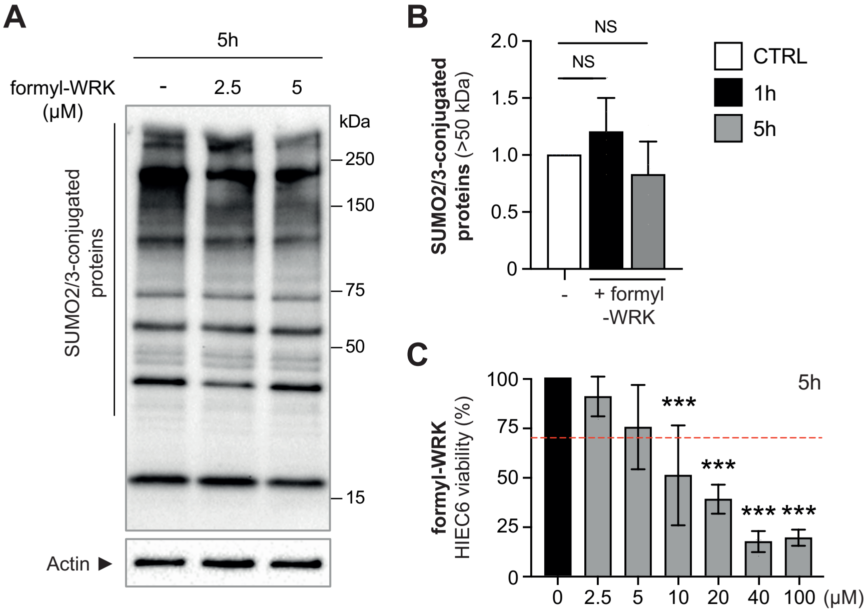

Figure S3: Lack of effect of Warnericin RK on SUMOylation in HIEC6 cells.

(A) Immunoblot analysis of SUMO2/3-conjugated proteins in HIEC6 cells treated with 2.5 or 5 µM formyl-WRK for 5h. (B) Quantification of SUMO2/3-conjugated proteins levels (above 50 kDa), after normalization by actin levels, in HIEC6 cells treated with 5 µM formyl-WRK for 1 or 5h. Values are expressed as fold-change versus untreated cells (mean ± s.d.; n=6; NS, not significant; One-way ANOVA, with Dunnett's correction). (C) Viability of HIEC6 cells incubated for 5h with increasing concentrations of formyl-WRK (mean ± s.d.; n=3-4; ***, P<0.001; One-way ANOVA, with Dunnett's correction).
